# Effects of degree of milling on nutritional quality, functional characteristics and volatile compounds of brown rice tea

**DOI:** 10.3389/fnut.2023.1232251

**Published:** 2023-08-24

**Authors:** Lei Zhou, Yong Sui, Zhenzhou Zhu, Shuyi Li, Rui Xu, Junren Wen, Jianbin Shi, Sha Cai, Tian Xiong, Fang Cai, Xin Mei

**Affiliations:** ^1^Key Laboratory of Agro-Products Cold Chain Logistics, Ministry of Agriculture and Rural Affairs, Institute of Agro-Products Processing and Nuclear-Agricultural Technology, Hubei Academy of Agricultural Science, Wuhan, China; ^2^School of Modern Industry for Selenium Science and Engineering, Wuhan Polytechnic University, National R&D Center for Se-Rich Agricultural Products Processing, Hubei Engineering Research Center for Deep Processing of Green Se-rich Agricultural Products, Wuhan, China

**Keywords:** brown rice tea, degree of milling, nutrients, aroma compounds, OPLS-DA

## Abstract

This study investigated the effects of rice preparation using different degrees of milling (DOM) from 0% to 13% on the nutritional composition, functional properties, major volatile compounds and safety of brown rice tea (BRT). We found that 2% DOM reduced 52.33% of acrylamide and 31.88% of fluorescent AGEs. When DOM was increased from 0% to 13%, the total phenolic content (TPC) of brown rice tea decreased by 48.12%, and the total flavonoid content (TFC) and condensed tannin content (CTC) also decreased significantly, with the smallest decrease at 2% DOM. In addition, the inhibitory activities of α-amylase, α-glucosidase and pancreatic lipase as well as the antioxidant activity also decreased gradually. Analysis by electronic nose and gas chromatography-mass spectrometry (GC-MS) showed that alkanes, furans, aldehydes, pyrazines and alcohols were the major volatiles in BRT, with 2% DOM having the greatest retention of aroma compounds. An orthogonal partial least squares discriminant analysis (OPLS-DA) and VIP score (VIP > 1 and *p* < 0.05) analysis were used to screen 25 flavor substances that contributed to the differences in BRT aroma of different DOMs. These results suggest that 2% milled BRT can improve safety and palatability while maximizing the retention of flavor compounds and nutrients. The findings of this study contribute to an enhanced understanding of the dynamics of changes and preservation of aroma compounds and nutrients present during the processing of BRT.

**Graphical Abstract:**
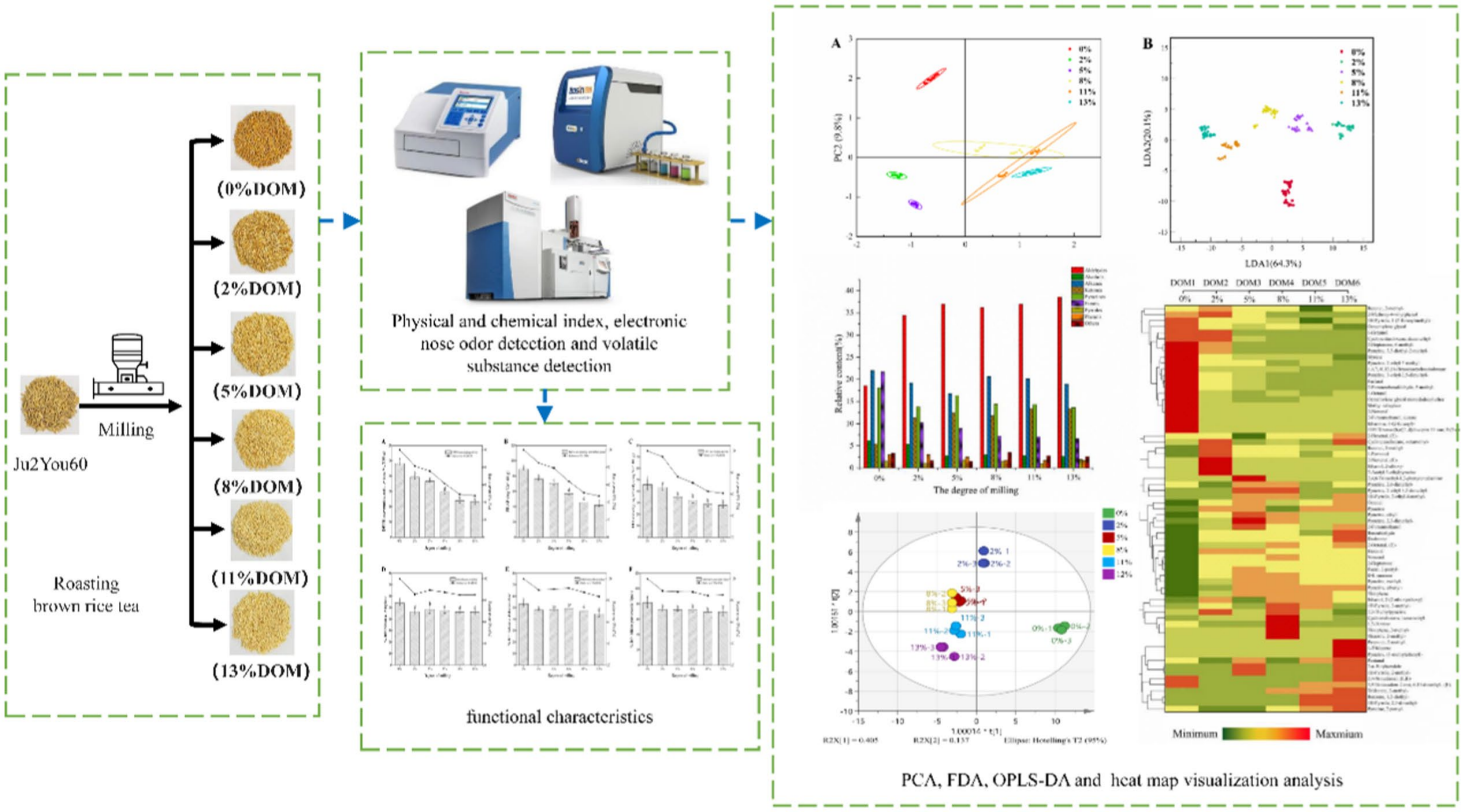


## 1. Introduction

Rice can be eaten on its own as a staple food or processed into products including cereal teas, noodles, wine, cookies, bread and condiments (1, 2). Among them, brown rice tea (BRT) is popular in East Asia because of its rich roasted flavor and probiotic effects (1). However, the edible quality of BRT made from brown rice is poor and hard in texture, which seriously affects the market value (3). DOM is a critical parameter that influences the physicochemical and organoleptic properties of rice. Previous studies have shown that higher DOM improves sensory quality and consumer acceptability, but leads to a decrease in the content of key nutrients, TPC and cellular antioxidant activity values in rice (4). Current studies have concentrated on the effects of DOM on phenolic compounds, physicochemical properties and functional properties of rice, but there are no relevant studies on BRT. Also, the effect of the DOM on acrylamide (AA) and advanced glycation end products (AGEs) in BRT has not been studied.

Furthermore, different DOMs can significantly affect the volatile compounds of rice due to the different concentrations of volatile compounds produced during the milling process (5). Previous studies have shown that the total volatiles of both rice and black rice were significantly lower after milling, but moderate milling resulted in better aroma characteristics and acceptability (5, 6). However, to date, few reports have investigated the effect of processing on the dynamics and small differences in the key aroma volatiles of BRT. Therefore, further research on proper BRT milling conditions is necessary to better understand the causes of flavor substance changes in BRT. This could provide theoretical support to improve the current BRT production technology and retain the aroma compounds in BRT.

Taking into account above factors, the security, physicochemical properties, phenolic compounds content and functional characteristics of BRT with different DOMs were investigated. Additionally, the effect of milling on BRT aroma compounds was analyzed by using a combination of electronic nose and GC–MS. Finally, the OPLS-DA model was used to gain insight into the correlation between the aroma volatility spectrum and the DOM. The conclusions will help to optimize the grinding conditions for BRT processing and provide a scientific basis for balancing the flavor quality and nutritional composition of BRT during the grinding process.

## 2. Materials and methods

### 2.1. Preparation of BRT

Rice of the indica cultivar Juliangyou-60 was supplied by the Food Crops Institute, Hubei Academy of Agricultural Sciences (Hubei Province, China). The brown rice was milled for 0 s, 1 s, 3 s, 6 s, 9 s and 12 s using the SXJMJ-858 rice milling machine (Zhejiang Taizhou Huachen Grain and Oil Machinery Co. Ltd., Zhejiang, China). Six different DOMs of rice (0, 2, 5, 8, 11 and 13% DOM) were obtained by the ratio of the weight of milled rice bran to the weight of unmilled brown rice. DOM is calculated according to Eq. (1) According to Nie et al. (7), BRT is produced by three sequential steps of soaking, drying, and roasting. Brown rice was soaked in water (1,10 w/v) for 10 min at room temperature. Approximately 100 g of drained brown rice was roasted at 200°C for 35 min, cooled, and collected and stored.


(1) $$DOM=1-\frac{\mathrm{weight of milled rice}}{\mathrm{weight of brown rice}}\times 100\%$$

### 2.2. Physicochemical characteristics

The atmospheric pressure drying method was used to determine the moisture content. AACC method 08–01 and method 86–80 were used to determine the content of ash, protein, lipid, and dietary fiber (DF) (8). The total starch content was established according to the procedure reported by Hongyan et al. (9). The content of reducing sugars (glucose) was determined by Zhiming et al. (8), with minor modifications. The color characteristics of BRT were measured with a CS-580B spectrophotometer (CHN Spec, LTD, China). The browning index (BI) and color difference (ΔE) were calculated by the literature equations (1, 10).

### 2.3. Determination of acrylamide and fluorescent AGEs

Acrylamide was analyzed in triplicate according to the method of Champrasert et al. (11), with slight modifications. An HPLC system equipped with an autosampler and UV detector (Waters e2695) was used. Two micro-open samples were injected with the autosampler and separated with an Iintersustain AQ-C18 analytical column (4.6 mm x 250 mm, 5 μm). The mobile phase was 0.8 mL/min of methanol (Sigma-Aldrich) (95% water). There was a run time of 10 min and a retention time of 6.73 min for acrylamide. A range of concentrations (0.1–2 μg/mL) of acrylamide solutions were prepared using acrylamide (Sigma-Aldrich. A9099). Using Milli-Q water, test samples were diluted, filtered through Nizhny Tsuji filters (0.45 μm) (Sigma-Aldrich), and then analyzed for acrylamide.

Fluorescent AGEs were obtained using a method described by Wang et al. (12), with slight modifications. A fluorescence spectrophotometer was used to measure the fluorescence intensity of AGEs by taking 2.0 g of rice tea powder (passed through 100 mesh sieve), adding it to 50 mL of neutral phosphate buffer solution and shaking it at 37°C for 4 h to dissolve the fluorescence substance as completely as possible, then centrifuging it at 4000 r/min for 10 min, taking the supernatant and fixing it to 50 mL, then taking 3 mL of the solution and a 370 nm excitation wavelength, a 440 nm emission wavelength, and a 5 nm slit are used. A measurement of the fluorescence of phosphate buffer solution was used as 1 custom unit (AUF), and the fluorescence intensity of the sample was (AU/g).

### 2.4. Determination of TPC, TFC, and CTC

The extraction of bound and free polyphenols from BRT was made according to the method described by Li et al. (13). Based on these reports, to determine the phenolic content, flavonoids and condensed tannins (14, 15).

### 2.5. Estimation of antioxidant capacity of BRT

#### 2.5.1. DPPH radical scavenging activity

The method for the DPPH assay was adapted from Fukui et al. (16). Briefly, 1.0 mL of rice tea extract and 2.0 mL of DPPH free radical ethanol solution (0.1 mM) were mixed well. A control was prepared by substituting 1.0 mL of aqueous methanol solution (40 mL/100 mL) for the extract. After mixing well, the mixture was left in the dark for 30 min and 517 nm absorbance measurement was conducted. Eq. (2) was used to calculate the radical scavenging activity of DPPH:


(2) $$\mathrm{DPPH radical scavenging activity}\;\left(\%\right)=\left(A_{c}-A_{s}\right)/A_{c}\times 100$$

where, Ac = Absorbance of control at 517 nm, As = Absorbance of the sample at 517 nm.

#### 2.5.2. Ferric reducing antioxidant power (FRAP) assay

Zhang et al. (17) reported a method for performing the FRAP assay.

#### 2.5.3. Assay for the scavenging effect on hydroxyl (OH) radicals

The method for the OH assay was adapted from Yongxu et al. (18). Briefly, the reaction mixture contained 1.0 mL of 9 mM salicylic acid-methanol solution, 1.0 mL of 9 mM FeSO4, 1.0 mL of 8.8 mM H2O2, and 1 mL of extract for the reaction. Fill to 10 mL with vial. Distilled water. After standing for 20 min, the absorbance of the mixture was determined at 510 nm. Eq. (3) was used to calculate the OH assay:


(3) $$\mathrm{OH}\;\mathrm{radical scavenging activity}\;\left(\%\right)=\left(A_{c}-A_{s}\right)/A_{c}\times 100\;$$

where, Ac = Absorbance of control at 624 nm, As = Absorbance of the sample at 624 nm.

### 2.6. Inhibitory properties against α-amylase, α-glucosidase and pancreatic lipase

#### 2.6.1. α-Amylase inhibition assay

The *α*-amylase inhibition assay was analyzed according to the method of Fatemeh et al. (19). Briefly, 0.3 mL of extract, 0.1 M sodium phosphate buffer (pH 6.8) in and 0.3 mL of 𝛼-amylase solution (1.0 U/mL) were mixed and set at 37°C for 10 min. After adding 0.3 mL of 1.0% (w/v) starch solution, it was again left to stand for 15 min., 0.5 mL of DNS was added and boiled for 8 min. After cooling, 517 nm absorbance measurement was conducted.

#### 2.6.2. α-Glucosidase inhibition assay

The 𝛼-glucosidase inhibition assay was analyzed according to the method of Zhang et al. (17), with some modifications. Briefly, 110 μL of PBS, 20 μL of extract and 20 μL of α-glucosidase (2.5 U/mL) were mixed and allowed to stand for 10 min at 37°C. Then, 20 μL of 1.25 mM pNPG was added and allowed to stand at 37°C for 20 min. 80 μL of 0.1 M Na_2_CO_3_ was added, followed by determination of absorbance at 405 nm.

#### 2.6.3. Pancreatic lipase inhibition assay

The pancreatic lipase inhibition assay was analyzed according to the method of Ruimin et al. (20). Briefly, 50 μL of extract and 50 μL of 25 U/mL pancreatic lipase were mixed and allowed to stand at 37°C for 10 min. Add 50 μL of 1.0 mg/mL of pNPB and leave at 37°C for 20 min, followed by determination of absorbance at 405 nm.

### 2.7. Electronic nose analysis

Using a slightly modified version of Yi et al.‘s (21) method, we performed e-nose analysis, with slight modifications. About 2 g of BRT, 10 mL of boiling water and 1.2 g of NaCl were added to a 20 mL headspace vial. After heating at 85°C for 30 min, an E-nose system (PEN3, AIRSENSE, Germany) was used. Acquisition at 50°C, 150 mL/min flow rate for 120 s and cleaning time for 120 s.

### 2.8. Volatile composition

With slight modifications, headspace solid-phase microextraction (HS-SPME) of the volatile components was conducted according to Yi et al. (21). Briefly, add 1 g BRT, 5 mL boiling water and 0.5 g NaCl to a 20 mL headspace vial. Stir in a water bath at 60°C for 20 min. The aged SPME fibers (DVB/CAR/PDMS fibers, Supelco Co., Bellefonte, PA, United States) were placed in the headspace of the sample vial for 30 min. The analysis of volatile aroma components was performed on an Agilent 7,890 gas chromatograph operated in combination with a 5975C triple quadrupole mass spectrometer (7890A/5975C, Agilent Technologies, Santa Clara, CA, United States). A DB-WAX capillary column (60 m × 250 μm × 0.25 μm) was used. See attached Supplementary data for parameter information.

### 2.9. Sensory evaluation of tea infusion

The sensory evaluation of rice tea was referenced from Hyun-Jin et al. (1) with slight modifications. Briefly, 25 g of complete BRT was boiled in 250 mL of boiling tap water for 3 min at a ratio of 1:10. The insulated tea infusion and rice grains were then separated and sensory evaluated. The sensory panel consisted of six male and six female members who were familiar with the principles and concepts of descriptive tea analysis. The evaluation included 5 aspects of aroma, flavor, color and overall acceptability/taste and was scored using a 9-point scale.

### 2.10. Statistical analysis

All data are averages obtained after repeating the experiment three times. Statistical analysis and graphical production of the data were performed by SPSS 26.0, Origin 2021, SIMCA-P version 14.1; and others the software was completed.

## 3. Results and discussion

### 3.1. Chromatic aberration

Color can greatly affect the quality of food and consumer acceptance (1). The color of BRT with different DOMs was presented in Table 1. There is a significant difference in the color, browning index and total color difference of BRT of different DOMs (*p* < 0.05). With the DOM of BRT increasing, the L* value increased, the a* and b* value decreased. These changes resulted in a decrease in the browning index (BI) of the BRT samples, indicating that grinding minimizes the browning of the samples. Similar patterns have been described for baked brown rice and roasted chickpeas (1, 22). Compared to BRT of milled, the 0% DOM BRT achieved maximum BI in the same baking time. This may be because 0% DOM BRT contains more free amino acids, reducing sugar and other non-enzymatic browning reaction substrate, which promoted the color reaction in the process of BRT roasting (1, 23). The color difference was relatively significant when DOM was more than 5%, suggesting that milling of brown rice significantly changes the color of BRT.

**Table 1:** Variation of acrylamide, fluorescent AGEs content and color of BRT with different DOMs.

DOMs	L*	a*	b*	Browning index	*ΔE*	Acrylamide(ug/kg)	Fluorescent AGEs(AU/g)
0%	72.07 ± 0.36^d^	4.86 ± 0.04^a^	17.60 ± 0.18^b^	32.45 ± 0.47^a^	–	1612.68 ± 171.56^a^	234.19 ± 8.01^a^
2%	75.50 ± 0.36^c^	4.12 ± 0.11^b^	17.35 ± 0.18^b^	29.63 ± 0.40^b^	3.52 ± 0.42^c^	768.79 ± 37.93^b^	159.54 ± 5.04^b^
5%	75.73 ± 0.76^c^	4.07 ± 0.12^b^	17.56 ± 0.26^b^	29.85 ± 0.81^b^	3.75 ± 1.11^c^	592.50 ± 27.43^b^	169.46 ± 7.32^b^
8%	77.49 ± 0.16^b^	3.20 ± 0.07^c^	16.49 ± 0.10^c^	26.50 ± 0.22^c^	5.78 ± 0.35^b^	699.93 ± 40.44^b^	146.41 ± 4.60^c^
11%	80.07 ± 0.46^a^	2.50 ± 0.038^d^	15.32 ± 0.21^d^	23.10 ± 0.36^d^	8.65 ± 0.77^a^	441.79 ± 29.14^c^	125.24 ± 3.12^d^
13%	80.70 ± 0.62^a^	2.49 ± 0.15^d^	16.15 ± 0.51^c^	24.14 ± 1.12^d^	9.08 ± 0.50^a^	432.57 ± 60.72^c^	117.71 ± 4.48^d^

The mean ± SD values in each column with different letters are significantly different (*p* < 0.05).

The Merad reaction produces toxic by-products such as AA and AGEs (12, 24). Notably, each milling stage resulted in a reduction of acrylamide and fluorescent AGEs content, which is not consistent with previous studies of milled chia seeds (24), AA and fluorescent AGEs are mainly formed in rice bran. Acrylamide content decreased by 52.33, 63.26, 56.60, 72.61 and 73.18%, respectively for brown rice when milled to 2, 5, 8, 11 and 13% DOM. The content of fluorescent AGEs decreased from 234.19 AU/g to 117.71 AU/g as DOM increased, with the largest decrease (31.88%) when DOM increased from 0 to 2%. This could be caused by the stripping of rice bran, which is enriched with reducing sugars, proteins and other substrates for the Merad reaction. This is consistent with the BI results.

### 3.2. Effect of DOM on the basic nutrients and phenolic compounds of BRT

As shown in Table 2 each grinding stage resulted in a decrease in water, lipids, protein, reducing sugars, dietary fiber and ash and an increased total starch, as previously reported (8). Dietary fiber content reduced to extent of 2.44, 2.29, 2.16, 0.70 and 0.69%, respectively for brown rice when milled to 0, 2, 5, 8 and 13% DOM. The reason for this phenomenon is that when rice is milled, the layer of rice bran containing dietary fiber is removed at the beginning (8). The reducing sugar content decreased from 0.85 to 0.67% with increasing DOM, with the greatest decrease (21.2%) when DOM increased from 0 to 2%. Reducing sugars are involved in heat-induced non-enzymatic browning reactions and their content defines the flavor, color and consumer satisfaction of baked goods (1). It is noteworthy that the moisture content decreased significantly from 2.15 to 1.20% with the increase in the DOM, which is inconsistent with the reports from the past (25). This may be due to the increase in DOM, which makes the water inside the rice more susceptible to participate in the browning reaction (26, 27). In addition, the lipid in BRT was reduced from 2.43 to 0.29%. Lipid oxidation can generate off-flavors, and this study found that milling processing significantly reduced lipids in BRT, which may inhibit the generation of off-flavors during BRT storage. The BRT without milling process contained 73.08% starch and reached a maximum starch content of 85.18% at 13% DOM, which was caused by the reduction in the proportion of other components.

**Table 2:** Basic nutrients of BRT of different DOMs.

DOMS	Moisture (g/100 g)	Lipid (g/100 g DW)	Protein (g/100 g DW)	Reducing sugars (g/100 g DW)	Dietary fiber (g/100 g DW)	Total starch (g/100 g DW)	Ash (g/100 g DW)
0%	2.15 ± 0.06^a^	2.43 ± 0.09^a^	9.22 ± 0.15^a^	0.85 ± 0.05^a^	2.44 ± 0.03^a^	73.08 ± 4.76^c^	1.05 ± 0.07^a^
2%	1.79 ± 0.03^a^	2.27 ± 0.24^b^	8.60 ± 0.14^b^	0.67 ± 0.04^b^	2.29 ± 0.09^ab^	74.40 ± 4.65^c^	0.81 ± 0.11^b^
5%	1.53 ± 0.04^a^	1.97 ± 0.40^c^	8.55 ± 0.21^bc^	0.58 ± 0.03^c^	2.16 ± 0.13^b^	78.21 ± 3.40^bc^	0.47 ± 0.17^c^
8%	1.49 ± 0.05^b^	1.32 ± 0.08^cd^	8.51 ± 0.02^bcd^	0.60 ± 0.05^bc^	0.70 ± 0.01^c^	80.82 ± 1.84^ab^	0.45 ± 0.08^c^
11%	1.44 ± 0.01^b^	1.09 ± 0.37^d^	8.22 ± 0.19^cd^	0.63 ± 0.04^bc^	0.68 ± 0.02^c^	83.44 ± 2.35^ab^	0.24 ± 0.08^cd^
13%	1.20 ± 0.03^c^	0.29 ± 0.09^e^	8.25 ± 0.23^d^	0.58 ± 0.03^c^	0.69 ± 0.03^c^	85.18 ± 0.60^a^	0.27 ± 0.09^d^

The mean ± SD values in each column with different letters are significantly different (*p* < 0.05).

Table 3 shows that polyphenols, flavonoids, and condensed tannins contained in BRT with different DOMs differ significantly (*p* < 0.05). A large proportion of the polyphenolic substances in the BRTs were in the free state, accounting for 67.2–84.76% of the TPC. 0% DOM had the greatest content of free and total phenols with 198.05 and 256.55 mg GAE/100 g. A 5% increase in DOM was observed, the TPC in BRT decreased slightly and then decreased substantially thereafter, in agreement with previous studies on the TPC of different bran fractions of black rice (17). Compared to 0% DOM, TPC decreased by 7.55, 8.54, 32.58, 45.27 and 48.12% for 2–13% DOM, respectively. Similarly, similar changes were observed in the flavonoid content of BRT. The proportion of flavonoids in the free state was the largest in the BRT of all DOMs, accounting for 76.05–88.95% of the total flavonoids. The TFC reached 114.44 mg CAE/100 g DW at 2% DOM, the highest at this point. Compared to the 2% DOM, the TFC of the 5 and 8% DOM decreased by 24.01 and 40.81%, and then the total flavonoid content of the 11 and 13% DOM decreased rapidly to 45.05 and 56.73%, respectively.

**Table 3:** The total content of free and bound phenolics, flavonoids, and condensed tannins in different DOM samples and their percentage contribution to the total.

DOM	Phenolics (mg GAE/100 g DW)			Flavonoids (mg CAE/100 g DW)			Condensed tannins (mg CAE/g DW)
	Free	Bound	Total	Free	Bound	Total	
0%	198.05 ± 6.59a^A^ (77.20)^B^	58.50 ± 4.95ab (22.80)	256.55 ± 14.55a	99.35 ± 3.95a (87.68)	13.96 ± 0.40c (12.32)	113.31 ± 3.76a	22.76 ± 0.96b
2%	177.37 ± 12.55b (74.78)	59.82 ± 3.95ab (25.22)	237.19 ± 21.84a	97.25 ± 4.21a (84.98)	17.19 ± 0.40b (15.02)	114.44 ± 4.47a	22.51 ± 1.73b
5%	157.70 ± 11.91b (67.20)	76.95 ± 6.04a (32.79)	234.65 ± 10.88a	66.14 ± 6.03b (76.05)	20.82 ± 0.81a (23.94)	86.96 ± 10.82b	29.54 ± 1.15a
8%	127.65 ± 5.88c (73.80)	45.32 ± 3.83b (26.20)	172.97 ± 15.37b	52.97 ± 1.29c (78.21)	14.76 ± 1.02c (21.79)	67.74 ± 0.73c	18.80 ± 0.38 dc
11%	117.51 ± 2.27c (83.68)	22.91 ± 1.95c (16.32)	140.42 ± 7.92c	53.38 ± 1.03c (84.88)	9.51 ± 0.81d (15.12)	62.89 ± 1.84c	19.19 ± 0.77c
13%	112.82 ± 5.13c (84.76)	20.28 ± 1.91c (15.24)	133.10 ± 5.25c	44.05 ± 2.04d (88.95)	5.47 ± 0.41e (11.05)	49.52 ± 1.23d	17.27 ± 0.38d

GAE, gallic acid equivalents; CAE, catechin equivalents. (A) The mean ± SD values in each column with different letters are significantly different (*p* < 0.05). (B) Values in parentheses indicate the percentage contribution to the total content.

With Table 3 we found little variation in the proportion of free phenols and free flavonoids possessed by the BRT of all DOMs. This is the same as the contribution of free phenols and flavonoids in black rice bran (17). It’s worth noting that 5% DOM had the highest bound phenolics, flavonoids and condensed tannins levels, which were 76.95 mg GAE/100 g DW, 20.82 and 29.54 mg CAE/100 g DW, respectively. Bound phenols are reported to be able to covalently bind to dietary fiber components and release them in a free state after high temperature processing (28, 29). In 0 to 5% DOM, dietary fiber content was significantly higher than in 8 to 13% (*p* < 0.05, Table 1) and the degree of browning reaction was significantly lower in 5% DOM then that in 0, 2% DOM (*p* < 0.05, Table 2), thus reduced the release of bound phenolics. This explains why BRT with 5% DOM has the most bound phenols, flavonoids and condensed tannins.

### 3.3. Antioxidant properties and inhibitory effect on enzyme of BRT with different DOMs

**Figure 1:**
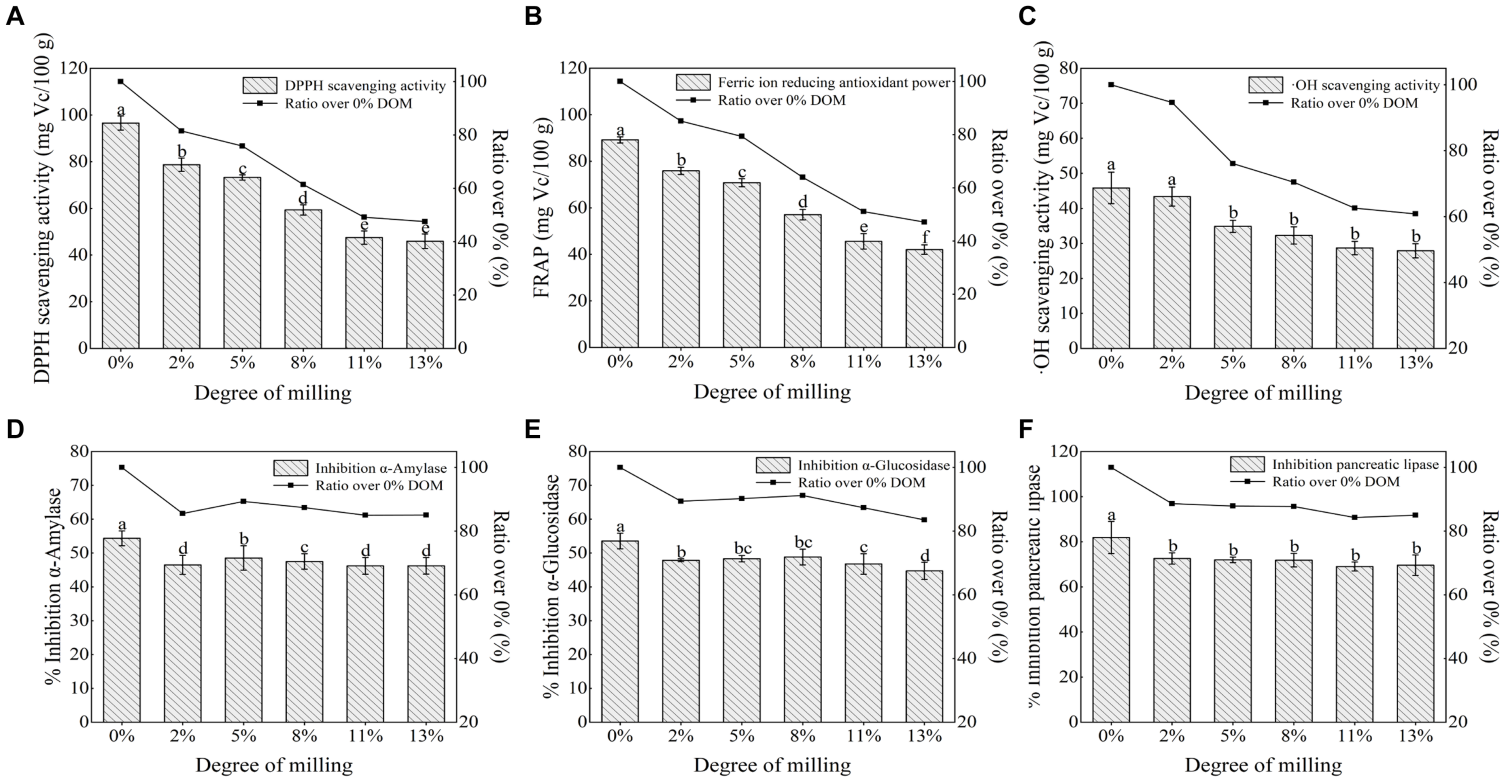
Comparison of **(A)** DPPH free radical scavenging activity, **(B)** Ferric ion reducing antioxidant power, **(C)** OH scavenging activity, **(D)** Inhibition α-amylase, **(E)** Inhibition α-glucosidase, **(F)** Inhibition Pancreatic lipase according to the DOM. Means with different letters in the same column are significantly different (*p* < 0.05).

As seen in Figures 1A–C, the extracts of each BRT showed better levels of antioxidant activity. 0% DOM possessed the highest DPPH, FRAP and OH value, which were 96.55 mg Vc/100 g DW, 89.18 mg Vc/100 g DW and 45.82 mg Vc/100 g DW, respectively. Compared to 0% DOM, the DPPH, OH radical scavenging and FRAP value of 2 to 13% DOMs decreased by 18.5, 24.1, 38.5, 50.8, 52.4%; 14.9, 20.6, 36.0, 48.9, 52.9%; and 5.3, 23.9, 29.6, 37.4, 39.2%, respectively. The 0% DOM rice tea underwent the strongest browning reaction after high temperature treatment, producing more dark pigments with antioxidant activity (30). In addition, the higher free and total phenolics levels may be reasons for the highest antioxidant capacity of the 0% DOM sample (31).

Regular consumption of cereal tea has a health effect of reducing the risk of type 2 diabetes (32, 33). The *α*-amylase and *α*-glucosidase inhibitory activities of 0% DOM samples (54.3 and 53.5%, respectively) were stronger than those of other samples (46.2–48.5% and 44.7–48. 8%, respectively), indicating that retention of whole rice bran significantly (*p* < 0.05) enhanced the *α*-amylase and *α*-glucosidase inhibitory activities (Figures 1D–F). The changes of the pancreatic lipase inhibitory activities in different DOMs were similar to those of the *α*-amylase and *α*-glucosidase inhibitory activities. The inhibition of pancreatic lipase decreased from 81.9 to 72.6% with increasing DOM, with the most significant decrease (11.4%) when DOM increased from 0 to 2%, and remained relatively stable thereafter. According to previous studies that brown rice is rich in ferulic acid, coumaric acid and apigenin, all of which have good inhibitory effects on the activities of alpha-amylase, alpha-glucosidase and pancreatic lipase (20, 34, 35). This also explains the significant ability of the 0% DOM sample to inhibit several enzyme activities mentioned above.

### 3.4. Electronic nose detection analysis of BRT with different DOMs

#### 3.4.1. Analysis of e-nose response values of BRT with different DOM

**Figure 2:**
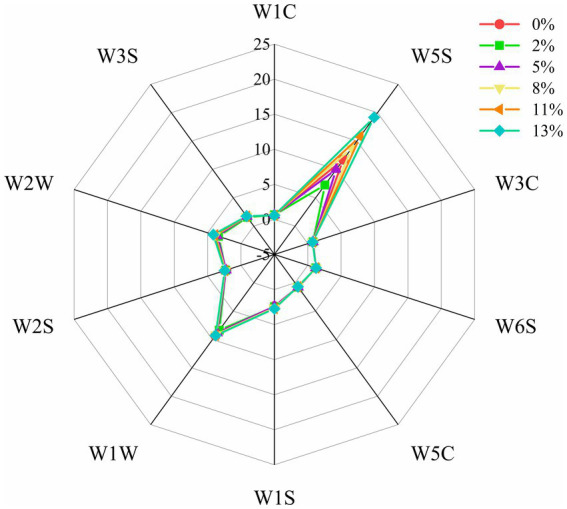
Radar fingerprinting of BRT volatile compounds under different DOMs.

As shown in Figure 2, the response intensity of sensors W1W, W1S, W2W, W3S, W2W and W6S were high and did not vary significantly, indicating that the inorganic sulfide, organic sulfide, long-chain alkanes, hydrogen, aromatic compounds and methyl components in BRT were high and DOM did not affect them significantly under different DOMs. The sensors W5S changed significantly, indicating that the length of the rice milling time had a significant effect on nitrogen oxides. The e-nose response values of BRT samples showed a tendency to decrease first with increasing DOM and then increase after 2% DOM. These results could also be related to the increase in DOM, which affected the content of free amino acids, lipids and reducing sugars in the bran layer, leading to a decrease in the degree of some non-enzymatic browning reactions, which ultimately affected the oxidative decomposition of alcohols and aldehydes (36, 37).

#### 3.4.2. Identification of BRT subjected to different DOMs using an electronic nose

**Figure 3:**
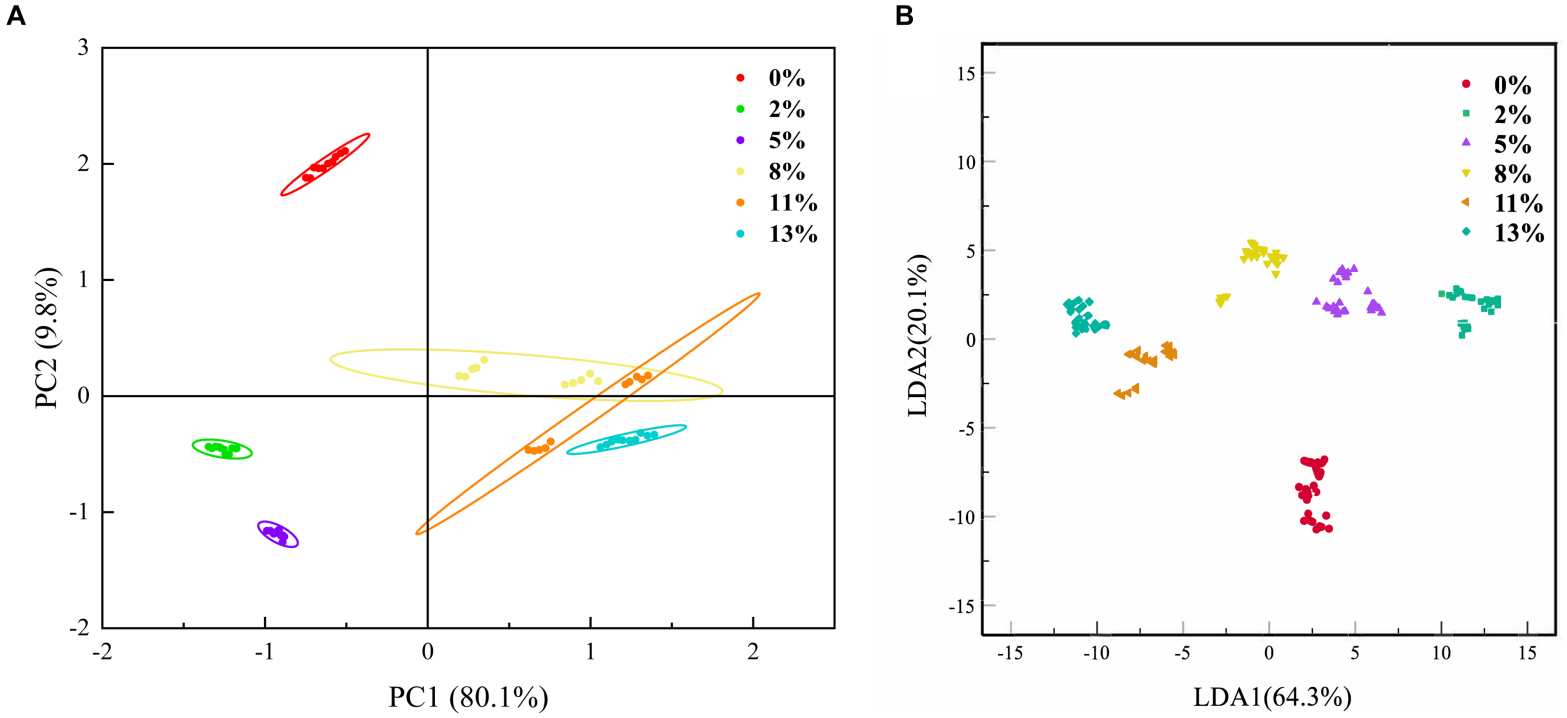
Electronic nose-based method for differentiation of BRT with different DOM. **(A)** Principal component analysis (PCA) and **(B)** Fisher discrimination analysis (FDA).

As in Figure 3, we combined PCA and FDA analysis to further distinguish the BRT volatile substance characteristics of different DOMs (37). The induction values from 95 s to 105 s were used as eigenvalues for the analysis. Contribution of the first principal component and the second principal of BRT with different DOMs were 80.1 and 9.8%, respectively, with a cumulative contribution of 89.9%, so samples can be analyzed largely based on these two principal components. 8, 11, and 13% of DOMs m-cha were concentrated in the first and fourth quadrants, which indicated that these BRT samples contained more hydrocarbons, ketones, and nitrogen oxides. The analysis showed that the contribution of LDA1 and LDA2 of BRTs with different DOMs was 64.3 and 20.1%, respectively, with a cumulative contribution of 84.4%. These results suggest that the combination of PCA and FDA can well distinguish the BRT of different DOMs. For the 0, 2 and 5% DOM BRT samples, the distances in the PCA and FDA charts were distant from the other samples, indicating that the milling process significantly altered the odor of BRT.

### 3.5. GC–MS analysis of BRT with different DOM

**Figure 4:**
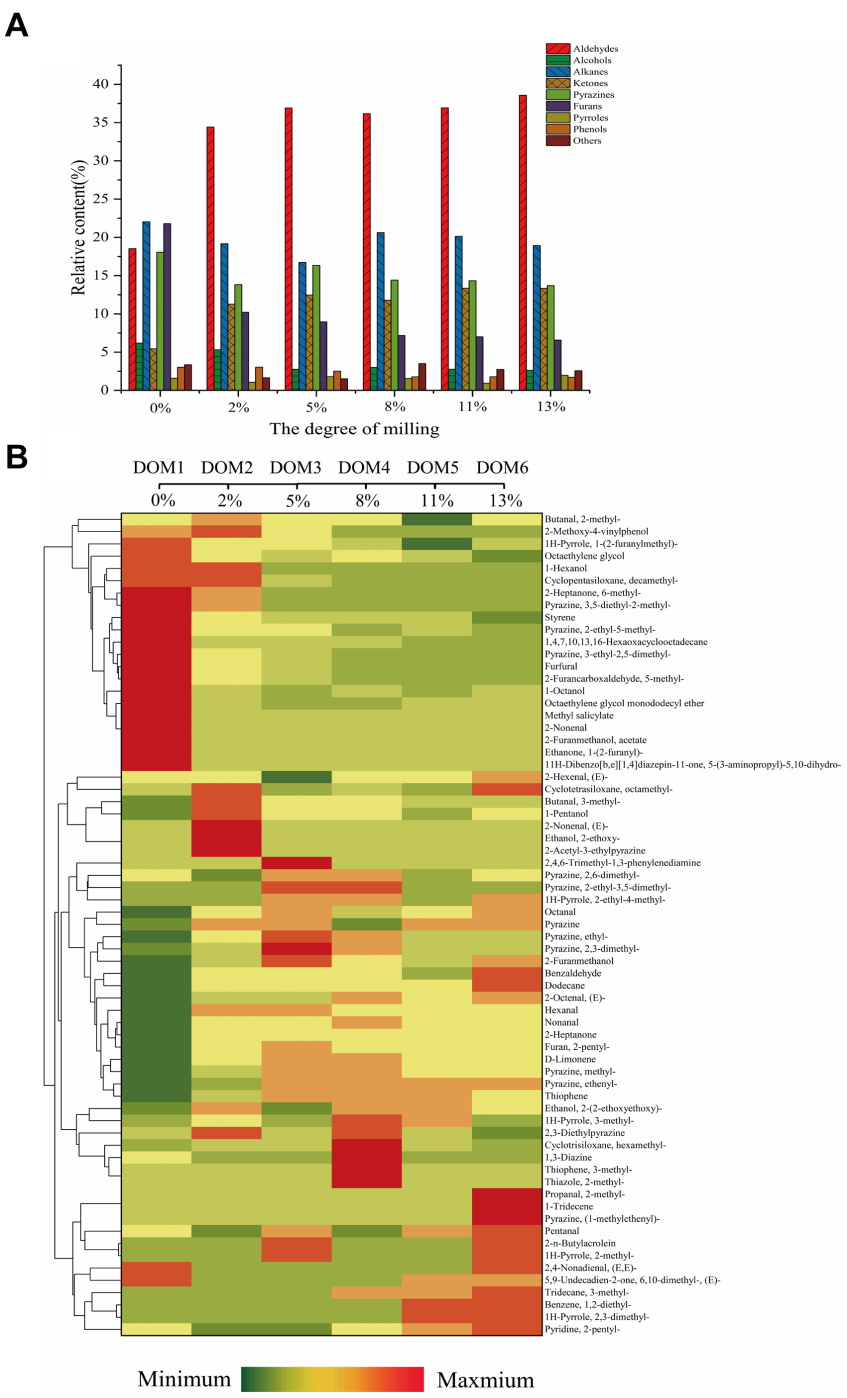
**(A)** Relative content of volatile substances of BRT with different DOM, **(B)** Clustering heat map of volatile compounds variation in BRT with different DOMs.

The BRT samples with different DOM (0, 2, 5, 8, 11 and 13%) contained a total of 67 volatile compounds classified into 17 groups including 14 aldehydes, 13 pyrazines, 5 furans, 5 pyrroles, 5 alcohols, 6 alkanes, 3 ketones, 3 alkenes, 2 esters, 2 ethers, 1 phenol, 1 benzene, 1 amine and 3 thiophenes. The results in Supplementary Table SA1 and Figure 4 show that alkanes, furans, aldehydes, pyrazines and alcohols were the most abundant volatiles in BRT (0% DOM) with ratios of 22.04, 21.80, 18.52, 18.07 and 6.20%, respectively. This is consistent with previous studies on baked brown rice (21). Among these volatile compounds, furans are the main providers of sweet and aromatic flavors, pyrazines provide strong roasted and nutty odors (38), and aldehydes and alcohols usually present herbaceous, green, floral and sweet flavors (39). With increasing grinding, the content of aldehydes and ketones increased about 1-fold, the content of furans, pyrazines and alcohols showed a decreasing pattern, while the content of alkanes, pyrroles and phenols remained unchanged.

As shown in Figure 4B, the key volatile compounds of BRT in the milling process were grouped into two clusters in the clustering heat map. DOM increased from 2 to 13% and hexanal, nonanal, benzaldehyde, 2-heptanone, pyrazine, methylpyrazine, 2-pentylfuran, 3-dimethyl-2,1H-pyrrole, thiophene, and 3-methyltridecane were increased compared to BRT without milling. Hexanal is formed by the hydrolyzed oxidation of fatty acids and is the dominant aroma in green tea (40). It has an extremely low odor threshold and usually provides an herbal aroma (41). Benzaldehyde is formed by Strecker degradation, which may impart a bitter perception (42). Pyrazine, methylpyrazine, 2-pentylfuran and 3-dimethyl-2,1 h-pyrrole provide roasted, nutty and floral flavors, which are desirable properties for baked goods. Nonanal is the main aroma contributor present in large quantities in unroasted BRT infusions and usually exhibits an off-flavor. It can be formed by the oxidation of oleic acid (1).

In the second group of volatiles, some of the aroma contributors of rice were ground off, like 2-ethyl-5-methylpyrazine, 3-ethyl-2,5-dimethylpyrazine, 3,5-diethyl-2-methylpyrazine, furfural, 5-methyl-2-furancarboxaldehyde, 1-(2-furanylmethyl)-1H-pyrrole, 1-octanol, (e)-2-nonenal, 6-methyl-2-heptanone, and styrene, resulting in a significant reduction in the aroma intensity of BRT. The reduction in the content of these aromas could be attributed to the removal of a large amount of non-enzymatic browning reaction substrates with the milling process (6). Furfural, 5-methyl-2-furancarboxaldehyde and 3-ethyl-2,5-dimethylpyrazine are the major furan and pyrazine volatiles in BRT. The reduction of these substances significantly reduced the roasted, cocoa and nutty aromas in the tea (43). Therefore, it can be concluded that the milling process significantly changes the concentration and diversity of flavor substances in BRT, which can significantly affect the flavor quality of BRT (5).

### 3.6. Descriptive discrimination of flavor substances of BRT during milling

**Figure 5:**
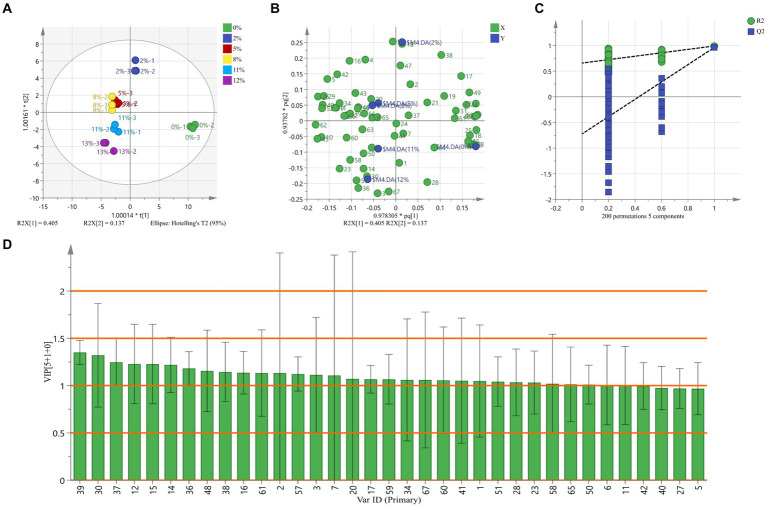
Descriptive discrimination of flavor substances of BRT during milling. **(A)** OPLS-DA results from GC–MS data of BRT at different milling stages. **(B)** The OPLS-DA score plot. **(C)** The result of 200 times permutation testing for OPLS-DA. **(D)** VIP values.

We analyzed the changes of the grinding process on the volatile compounds of BRT by building an OPLS-DA model. OPLS-DA uses orthogonal signal correction to select signals that are strongly associated with the model classification, better separating samples between groups and making the explanatory power clearer and more credible (44). As shown in the OPLS-DA score plot in Figure 5A, the first and second components accounted for 40.5 and 13.7% of the total variables, respectively (R2Xcum = 54.2%). The results can reflect the greater the difference in the relative positions of BRTs with different DOMs in the score plot (Figure 5B). Based on the OPLS-DA model, it showed a good fit (R2Ycum = 0.962) and prediction (Q2cum = 0.782) for the identification of flavor substances in BRT during the milling process. R2 and Q2 exceeding 0.5 indicates that model fitting results are acceptable (45). We have verified it using interchange tests. To verify whether the model has overfitting problems, we performed 200 iterations of the model (R2, Q2 intercepts were 0.657 and − 0.722) (Figure 5C). It can be seen that this model is able to differentiate well between samples. The predicted variable importance (VIP) values can be derived from the PLS regression model for the flavor substances of BRT during milling processing (Figure 5D). The VIP scores for 2-methyl-thiazole, 2-pentyl-pyridine, 2-ethyl-4-methyl-1 h-pyrrole, 2-methyl-1 h-pyrrole, 2,3-dimethyl-1 h-pyrrole, 2-acetyl-3-ethylpyrazine, (1-methylethenyl)-pyrazine, 2-ethyl-3,5-dimethyl-pyrazine, 2,6-dimethyl-pyrazine, 2,4,6-trimethyl-1,3-phenylenediamine, 1,2-diethyl-benzene, 2-(2-ethoxyethoxy)-ethanol, 1-tridecene, 6,10-dimethyl-(e)-5,9-undecadien-2-one, 3-methyl-tridecane, 1-hexanol, 1-pentanol, 2-ethoxy-ethanol, (E,E)-2,4-nonadienal, (E)-2-nonenal, (E)-2-hexenal, pentanal, 2-methyl-butanal and 2-methyl-propanal were > 1. These compounds can be used as an indicator to identify the degree of BRT milling (VIP > 1 and *p* < 0.05).

### 3.7. Sensory evaluation of BRT tea infusion and rice grains

In this study, sensory evaluation of tea infusion and rice grains from BRT made from rice with different DOMs was carried out (Table 4). The BRT samples with different DOM exhibited significant differences in color, aroma, flavor, texture and overall acceptability (*p* < 0.05). With the DOM of BRT increasing, the overall acceptance of tea infusions decreased, the mouthfeel score of rice grains decreased. This is because 0% DOM BRT contains the most dietary fiber, resulting in greater hardness and poorer taste of rice grains (46). Because of the reduced browning reaction, DOM revealed a negative correlation with the color and taste of BRT. With the higher DOM had greater intensities of sweet taste (46), so the aroma score revealed a positive correlation with DOM. In conclusion, the degree of milling of BRT can significantly change the sensory characteristics (color, texture and taste) and overall acceptability of tea infusion and rice grains. A proper level of grinding can usefully enhance the overall sensory quality and acceptance of the BRT.

**Table 4:** Sensory characteristics of tea infusion and rice grains from BRT samples produced by different DOM.

Samples	DOMs	Color	Aroma	Taste	Overall acceptance/ Mouthfeel
Tea infusions	0%	7.61 ± 0.44^a^	4.69 ± 0.36^a^	7.25 ± 0.58^a^	6.44 ± 0.31^a^	2%	6.93 ± 0.44^b^	5.83 ± 0.24^b^	6.56 ± 0.25^b^	5.83 ± 0.55^ab^	5%	5.55 ± 0.38^c^	5.48 ± 0.36^bc^	5.49 ± 0.33^c^	5.74 ± 0.60^b^	8%	5.18 ± 0.43^cd^	5.83 ± 0.48^bc^	4.82 ± 0.35^d^	6.08 ± 0.61^b^	11%	4.93 ± 0.40^d^	6.08 ± 0.68^c^	4.28 ± 0.29^e^	5.69 ± 0.33^b^	13%	3.93 ± 0.29^e^	6.99 ± 0.36^d^	3.54 ± 0.26^f^	5.94 ± 0.57^b^
Rice grains	0%	7.26 ± 0.38^a^	4.01 ± 0.38^c^	4.68 ± 0.34^d^	2.57 ± 0.37^e^	2%	6.45 ± 0.36^b^	4.16 ± 0.33^c^	5.58 ± 0.30^c^	4.49 ± 0.62^d^	5%	5.39 ± 0.49^c^	4.62 ± 0.37^b^	5.95 ± 0.60^b^	5.73 ± 0.57^c^	8%	5.08 ± 0.73^c^	6.27 ± 0.49^a^	6.65 ± 0.35^a^	6.62 ± 0.34^b^	11%	4.28 ± 0.38^d^	6.33 ± 0.59^a^	5.84 ± 0.35^bc^	7.33 ± 0.58^a^	13%	3.75 ± 0.60^e^	6.27 ± 0.64^a^	5.68 ± 0.36^bc^	6.69 ± 0.57^b^

Means with different letters in the same column are significantly different (*p* < 0.05).

## 4. Conclusion

Our study investigated the effects of different DOMs on BRT essential nutrients, phenolics, their distribution, biological activity, and volatile compound content. Acrylamide and AGEs were effectively reduced at 2% DOM. The 0% DOM samples contained noticeably higher TPC, TFC and CTC and showed stronger antioxidant and enzyme inhibitory activities compared to other DOM samples. Alkanes, furans, aldehydes, pyrazines and alcohols are the main volatile compounds in BRT. It can be concluded from the Herarchical clustering and OPLS-DA analysis that 2% DOM is the optimal milling condition, as this condition retains most of the aromatic compounds and nutrients, and the safety and taste are greatly enhanced. Finally, twenty-five aroma-active compounds may have contributed to rice type discrimination, like 1-tridecene, 2,4,6-trimethyl-1,3-phenylenediamine, (E)-2-nonenal, (E, E)-2,4-nonadienal and 2-ethoxy-ethanol. This study provides significant data to guide the appropriate processing and aroma quality of BRT.

## Data availability statement

The original contributions presented in the study are included in the article/Supplementary material, further inquiries can be directed to the corresponding authors.

## Author contributions

LZ: methodology, investigation, and writing. YS: investigation and funding acquisition. ZZ: investigation. SL: funding acquisition and writing – original draft. RX: writing – original draft. JW: investigation and visualization. JS: investigation. SC and TX: supervision and resources. FC: supervision, funding acquisition, and resources. XM: conceptualization, supervision, funding acquisition, and writing – review & editing. All authors contributed to the article and approved the submitted version.

## Funding

This work was supported by the project of Special Project for Science and Technology Innovation of Wuhan (2022020801020344). Science and Technology Plan Project of Hubei Province (2023BBB101). The support of the National Key Research and Development Program of China (2018YFD0301306-4-2), Outstanding young and middle-aged science and technology innovation team in Hubei Province (T2020012), the Science and Technology Plan Project in Agriculture and Rural Areas of Hubei Province in 2023 were also appreciated.

## Conflict of interest

The authors declare that the research was conducted in the absence of any commercial or financial relationships that could be construed as a potential conflict of interest.

## Publisher’s note

All claims expressed in this article are solely those of the authors and do not necessarily represent those of their affiliated organizations, or those of the publisher, the editors and the reviewers. Any product that may be evaluated in this article, or claim that may be made by its manufacturer, is not guaranteed or endorsed by the publisher.
