# Supplementary material for: Effects of degree of milling on nutritional quality, functional characteristics and volatile compounds of brown rice tea
Source: Front Nutr. 2023 Aug 24;10:1232251. doi: 10.3389/fnut.2023.1232251 (PMC10483151; doi:10.3389/fnut.2023.1232251)
Supplement: Supplementary file 1 [file Data_Sheet_1.docx]

Supplementary Material

**Effects of degree of milling on the phenolic contents, functional characteristics and volatile compounds of brown rice tea**

Lei Zhou^1,2^, Yong Sui^1*^, Zhenzhou Zhu^2^, Shuyi Li^2^, Rui Xu^1,2^, Junren Wen^1^, Jianbin Shi^1^, Sha Cai^1^, Tian Xiong^1^, Fang Cai^1^, Xin Mei^1*^

*** Correspondence:**
Yong Sui, E-mail address: suiyong0429@foxmail.com. Tel Number: +86-27-87389307

Xin Mei, E-mail address: liangshijiagong@126.com. Tel Number: +86-27-87389307

# Supplementary methods

## GC-MS detailed parameters

The analysis of volatile aroma components was performed on an Agilent 7890 gas chromatograph operated in combination with a 5975C triple quadrupole mass spectrometer (7890A/5975C, Agilent Technologies, Santa Clara, CA, USA). A DB-WAX capillary column (60 m × 250 μm × 0.25 μm) was used. The injector temperature was maintained at 250°C. High purity helium was used as the carrier gas at a flow rate of 1.0 mL/min. The oven temperature was set at 30°C for 1 min, ramped up to 210°C at 7°C/min, held at 150°C for 2 min, and finally ramped up to 250°C at 5°C/min and held isothermally for 3 min. The electron ionization source temperature was kept at 230 °C, and the mass spectra were obtained by an electron impact of 70 eV. The interface and quadrupole temperatures were 280 °C and 150 °C, respectively. Data were acquired in full scan mode in the range of 25-450 amu.

# Supplementary Figures and Tables

## Supplementary Table

**Table A1**

Area ratio of volatile compounds in tea infusions prepared from brown rice tea prepared by different degree of milling.

| NO | Category | RT（min） | Component name | Odor description | Area ratio |  |  |  |  |  |
| --- | --- | --- | --- | --- | --- | --- | --- | --- | --- | --- |
|  |  |  |  |  | DOM 0% | DOM 2% | DOM 5% | DOM 8% | DOM 11% | DOM 13% |
| 1 | Aldehydes | 6.551 | 2-methyl-propanal |  | ND | ND | ND | ND | ND | 0.266 ± 0.065 |
| 2 |  | 8.879 | 2-methyl-butanal |  | 0.498 ± 0.166a | 0.534 ± 0.007a | 0.523 ± 0.027a | 0.516 ± 0.029a | ND | 0.421 ± 0.048a |
| 3 |  | 10.658 | pentanal | cheesy, sour | 0.438 ± 0.013c | ND | 0.670 ± 0.039b | ND | 0.608 ± 0.001b | 0.764 ± 0.030a |
| 4 |  | 10.704 | 3-methyl-butanal | almond, apple, acrid | 0.357 ± 0.016b | 0.773 ± 0.215a | 0.574 ± 0.059ab | 0.627 ± 0.044ab | 0.529 ± 0.029ab | 0.506 ± 0.061ab |
| 5 |  | 13.524 | hexanal | green, grassy, tomato | 2.791 ± 0.198b | 7.303 ± 0.163a | 7.140 ± 0.770a | 6.968 ± 0.559a | 6.236 ± 0.178a | 6.408 ± 0.347a |
| 6 |  | 15.472 | 2-n-butylacrolein |  | ND | ND | 0.102 ± 0.005a | ND | ND | 0.108 ± 0.009a |
| 7 |  | 17.041 | (E)-2-hexenal | grassy | 0.079 ± 0.001ab | 0.082 ± 0.000ab | ND | 0.085 ± 0.000a | 0.073 ± 0.001b | 0.089 ± 0.007a |
| 8 |  | 18.607 | octanal | citrus | 0.998 ± 0.093a | 1.085 ± 0.055a | 1.120 ± 0.102a | 1.061 ± 0.088a | 1.093 ± 0.048a | 1.103 ± 0.093a |
| 9 |  | 20.818 | nonanal | citrus, floral | ND | 3.574 ± 0.073a | 3.943 ± 0.118a | 4.014 ± 0.159a | 3.654 ± 0.111a | 3.929 ± 0.251a |
| 10 |  | 21.618 | (E)-2-octenal | green, nut, fat | 0.405 ± 0.017c | 0.663 ± 0.036b | 0.720 ± 0.086b | 0.888 ± 0.053ab | 0.797 ± 0.014a | 0.913 ± 0.007a |
| 11 |  | 23.595 | benzaldehyde | nutty, sweet, almond | 1.364 ± 0.067c | 1.763 ± 0.022ab | 1.741 ± 0.059ab | 1.719 ± 0.064ab | 1.572 ± 0.037b | 1.895 ± 0.103a |
| 12 |  | 23.699 | (E)-2-nonenal | green, citrus | ND | 0.321 ± 0.001 | ND | ND | ND | ND |
| 13 |  | 23.702 | 2-nonenal | beany, cucumber | 0.427 ± 0.091 | ND | ND | ND | ND | ND |
| 14 |  | 26.942 | (E, E)-2,4-nonadienal | waxy | 0.158 ± 0.000b | ND | ND | ND | ND | 0.203 ± 0.007a |
| 15 | Alcohols | 17.138 | 2-ethoxy-ethanol |  | ND | 0.158 ± 0.000 | ND | ND | ND | ND |
| 16 |  | 17.69 | 1-pentanol | green | 0.300 ± 0.015c | 0.513 ± 0.006a | 0.427 ± 0.005b | 0.432 ± 0.007b | 0.357 ± 0.003c | 0.436 ± 0.042b |
| 17 |  | 19.894 | 1-hexanol | green, fruity, apple-skin, oily | 0.768 ± 0.018b | 0.840 ± 0.020a | ND | ND | ND | ND |
| 18 |  | 23.904 | 1-octanol | fruity, floral | 0.969 ± 0.042a | 0.689 ± 0.004b | 0.603 ± 0.025bc | 0.647 ± 0.025bc | 0.580 ± 0.024c | 0.680 ± 0.030b |
| 19 |  | 36.882 | octaethylene glycol |  | 0.424 ± 0.087a | 0.286 ± 0.200ab | 0.208 ± 0.057ab | 0.243 ± 0.021ab | 0.158 ± 0.044ab | 0.018 ± 0.010b |
| 20 | Alkanes | 7.474 | hexamethyl-cyclotrisiloxane |  | 3.302 ± 0.058b | 3.667 ± 0.156ab | 3.476 ± 0.338b | 4.628 ± 0.520a | 3.613 ± 0.333ab | 3.403 ± 0.325b |
| 21 |  | 14.977 | decamethyl-cyclopentasiloxane |  | 1.482 ± 0.057a | 1.438 ± 0.392a | 1.177 ± 0.350a | 1.031 ± 0.347a | 1.097 ± 0.217a | 1.004 ± 0.109a |
| 22 |  | 16.356 | dodecane | gasoline-like | 0.090 ± 0.002b | 0.270 ± 0.002a | 0.292 ± 0.003a | 0.282 ± 0.007a | 0.273 ± 0.007a | 0.356 ± 0.010a |
| 23 |  | 20.067 | 3-methyl-Tridecane |  | ND | ND | ND | 0.196 ± 0.001b | 0.228 ± 0.002b | 0.276 ± 0.019a |
| 24 |  | 21.13 | octamethyl-cyclotetrasiloxane |  | 2.914 ± 0.538a | 3.391 ± 0.450a | 2.670 ± 0.854a | 2.825 ± 0.649a | 2.715 ± 0.736a | 3.405 ± 0.836a |
| 25 |  | 37.596 | 1,4,7,10,13,16-hexaoxacyclooctadecane |  | 0.968 ± 0.017a | 0.201 ± 0.082b | 0.169 ± 0.037bc | 0.091 ± 0.004bcd | 0.014 ± 0.003d | 0.059 ± 0.022cd |
| 26 | Ketones | 16.137 | 2-heptanone | cheesy, Banana, cinnamon | 1.769 ± 0.068b | 5.157 ± 0.032a | 5.291 ± 0.405a | 5.172 ± 0.226a | 5.125 ± 0.191a | 5.218 ± 0.299a |
| 27 |  | 17.452 | 6-methyl-2-heptanone | cheesy | 0.210 ± 0.002a | 0.116 ± 0.009b | ND | ND | ND | ND |
| 28 |  | 29.745 | 6,10-dimethyl-(E)-5,9-undecadien-2-one | fresh, green, fruity, waxy | 0.178 ± 0.019a | ND | ND | ND | 0.140 ± 0.010a | 0.168 ± 0.004a |
| 29 | Alkenes | 16.522 | d-Limonene | lemon, orange | 0.081 ± 0.009c | 0.168 ± 0.014b | 0.199 ± 0.009ab | 0.209 ± 0.007a | 0.174 ± 0.016b | 0.169 ± 0.000b |
| 30 |  | 17.405 | 1-tridecene | gasoline-like to odourless | ND | ND | ND | ND | ND | 0.052 ± 0.006 |
| 31 |  | 18 | styrene | Sweet, balsamic, almost  floral odor | 0.279 ± 0.054a | 0.201 ± 0.003ab | 0.180 ± 0.033b | 0.166 ± 0.023b | 0.173 ± 0.022b | 0.136 ± 0.018b |
| 32 | Esters | 23.536 | 2-furanmethanol, acetate |  | 0.181 ± 0.002 | ND | ND | ND | ND | ND |
| 33 |  | 28.571 | methyl salicylate |  | 0.106 ± 0.019 | ND | ND | ND | ND | ND |
| 34 | Ethers | 25.286 | 2-(2-ethoxyethoxy)-ethanol |  | ND | 0.257 ± 0.008ab | ND | 0.306 ± 0.026a | 0.248 ± 0.001b | 0.235 ± 0.014b |
| 35 |  | 37.52 | octaethylene glycol monododecyl ether |  | 0.299 ± 0.086a | 0.026 ± 0.010b | ND | ND | 0.031 ± 0.000b | 0.016 ± 0.005b |
| 36 | Benzenes | 19.143 | 1,2-diethyl-benzene |  | ND | ND | ND | ND | 0.107 ± 0.006a | 0.121 ± 0.004a |
| 37 | Amines | 23.360 | 2,4,6-trimethyl-1,3-phenylenediamine |  | ND | ND | 0.076 ± 0.002 | ND | ND | ND |
| 38 | Phenols | 36.148 | 2-methoxy-4-vinylphenol | dry, woody, fresh, roasted | 1.196 ± 0.067b | 1.420 ± 0.080a | 1.126 ± 0.043b | 0.781 ± 0.025c | 0.708 ± 0.001c | 0.736 ± 0.045c |
| 39 | Pyrazines | 16.936 | pyrazine | pungent, roasted hazelnut, roasted barley, sweetcorn | ND | 0.293 ± 0.005a | 0.337 ± 0.008a | ND | 0.317 ± 0.002a | 0.299 ± 0.026a |
| 40 |  | 18.233 | methyl-pyrazine | nutty, roasted, cocoa and peanut-like- | 0.941 ± 0.004d | 1.309 ± 0.044c | 1.631 ± 0.005a | 1.563 ± 0.020ab | 1.435 ± 0.037abc | 1.417 ± 0.144bc |
| 41 |  | 19.623 | 2,6-dimethyl-pyrazine | nutty, coffee, cocoa-like | 0.813 ± 0.308a | 0.489 ± 0.024a | 0.925 ± 0.145a | 0.909 ± 0.157a | 0.571 ± 0.019a | 0.756 ± 0.061a |
| 42 |  | 19.744 | ethyl-pyrazine | nutty, coffee, cocoa-like | 1.022 ± 0.006c | 1.393 ± 0.051ab | 1.549 ± 0.028a | 1.421 ± 0.015ab | 1.289 ± 0.059b | 1.286 ± 0.133b |
| 43 |  | 20.019 | 2,3-dimethyl-pyrazine | nutty, cocoa-like | 0.336 ± 0.031b | 0.370 ± 0.014ab | 0.429 ± 0.008a | 0.395 ± 0.000ab | 0.375 ± 0.011ab | 0.363 ± 0.037ab |
| 44 |  | 20.956 | 2-ethyl-5-methyl-pyrazine | nutty, caramelic-like, coffee-like | 0.851 ± 0.019a | 0.648 ± 0.038b | 0.610 ± 0.026bc | 0.500 ± 0.025c | 0.546 ± 0.022bc | 0.485 ± 0.083c |
| 45 |  | 21.834 | ethenyl-pyrazine |  | 0.162 ± 0.000c | 0.245 ± 0.004b | 0.350 ± 0.012a | 0.349 ± 0.008a | 0.357 ± 0.016a | 0.353 ± 0.020a |
| 46 |  | 21.982 | 3-ethyl-2,5-dimethyl-pyrazine | nutty, hazelnut | 2.442 ± 0.150a | 1.178 ± 0.078b | 0.913 ± 0.120bc | 0.636 ± 0.155c | 0.672 ± 0.123c | 0.528 ± 0.175c |
| 47 |  | 22.17 | 2,3-diethylpyrazine |  | 0.093 ± 0.001a | 0.106 ± 0.010a | 0.092 ± 0.011a | 0.107 ± 0.007a | 0.090 ± 0.006a | 0.078 ± 0.013a |
| 48 |  | 22.317 | 2-ethyl-3,5-dimethyl-pyrazine | cocoa-like, coffee | ND | ND | 0.476 ± 0.003a | 0.446 ± 0.043a | ND | ND |
| 49 |  | 22.936 | 3,5-diethyl-2-methyl-pyrazine | cocoa-like, coffee | 0.519 ± 0.036a | 0.342 ± 0.025b | ND | ND | ND | ND |
| 50 |  | 22.992 | (1-methylethenyl)-pyrazine |  | ND | ND | ND | ND | ND | 0.327 ± 0.018 |
| 51 |  | 23.361 | 2-acetyl-3-ethylpyrazine |  | ND | 0.093 ± 0.003 | ND | ND | ND | ND |
| 52 | Furans | 17.249 | 2-pentyl-furan, | floral, fruit | 0.446 ± 0.024b | 0.822 ± 0.052a | 0.904 ± 0.104a | 0.889 ± 0.059a | 0.884 ± 0.007a | 0.894 ± 0.068a |
| 53 |  | 22.238 | furfural | sweet, woody, almond, bread baked | 6.208 ± 0.359a | 2.731 ± 0.084b | 1.859 ± 0.125c | 1.212 ± 0.050d | 0.919 ± 0.037d | 0.931 ± 0.079d |
| 54 |  | 23.11 | 1-(2-furanyl)-ethanone | sweet, almond, nutty | 0.233 ± 0.001 | ND | ND | ND | ND | ND |
| 55 |  | 24.444 | 5-methyl-2-furancarboxaldehyde | caramellic, sweet, coffee-like | 1.065 ± 0.103a | 0.446 ± 0.019b | 0.362 ± 0.024b | 0.221 ± 0.001c | 0.161 ± 0.008c | 0.165 ± 0.022c |
| 56 |  | 25.894 | 2-furanmethanol | burnt, sweet, caramellic | 0.706 ± 0.054b | 0.780 ± 0.020ab | 0.888 ± 0.029a | 0.829 ± 0.012ab | 0.797 ± 0.052ab | 0.840 ± 0.057ab |
| 57 | Pyrroles | 16.823 | 2,3-dimethyl-1H-pyrrole |  | ND | ND | ND | ND | 0.135 ± 0.005a | 0.129 ± 0.013a |
| 58 |  | 24.281 | 2-methyl-1H-pyrrole | nutty, sweet | ND | ND | 0.266 ± 0.002a | ND | ND | 0.310 ± 0.025a |
| 59 |  | 24.283 | 3-methyl-1H-pyrrole | nutty, sweet | ND | 0.172 ± 0.010b | ND | 0.280 ± 0.005a | 0.235 ± 0.050ab | ND |
| 60 |  | 26.992 | 2-ethyl-4-methyl-1H-pyrrole |  | ND | ND | 0.155 ± 0.001a | 0.149 ± 0.008a | ND | 0.143 ± 0.010a |
| 61 |  | 29.325 | 1-(2-furanylmethyl)-1H-pyrrole | green, waxy, fruity, coffee-like | 0.631 ± 0.124a | 0.330 ± 0.102b | 0.394 ± 0.017b | 0.254 ± 0.009b | ND | 0.275 ± 0.015b |
| 62 | Thiophenes | 11.897 | thiophene | alliaceous, garlic, sulfurous | ND | 0.120 ± 0.004b | 0.225 ± 0.010a | 0.224 ± 0.004a | 0.211 ± 0.019a | 0.203 ± 0.008a |
| 63 |  | 13.789 | 3-methyl-thiophene | alliaceous, onion, sulfurous | ND | ND | ND | 0.195 ± 0.044 | ND | ND |
| 64 |  | 32.255 | 5-(3-aminopropyl)-5,10-dihydro-11H-dibenzo[b,e][1,4]diazepin-11-one |  | 0.178 ± 0.024 | ND | ND | ND | ND | ND |
| 65 | Pyrimidines | 16.922 | 1,3-diazine |  | 0.081 ± 0.003a | ND | ND | 0.311 ± 0.010b | ND | ND |
| 66 | Thiazoles | 17.607 | 2-methyl-thiazole |  | ND | ND | ND | 0.360 ± 0.003 | ND | ND |
| 67 | Pyridines | 24.553 | 2-pentyl-pyridine |  | 0.123 ± 0.003b | ND | ND | 0.130 ± 0.015b | 0.138 ± 0.009b | 0.174 ± 0.001a |

RI: The retention index of volatile compounds on DB-WAX column.

Values with different letters (a–e) in a row are significantly different using Duncan’s multiple comparison tests (*p* < 0.05).

Odour descriptions were from the literatures (Chen et al., 2021; Sehun & Jihyun, 2021; Zhan, Liu, Su, Lin, & Ni, 2023), or from FEMA database.

ND: Not detected in sample.

**References**

Chen, G., Yan, L., Qifeng, P., Mingcong, F., Li, W., & Haifeng, Q. (2021). Analysis of the key aroma volatile compounds in rice bran during storage and processing via HS-SPME GC/MS. *Journal of Cereal Science, 99*. doi: 10.1016/j.jcs.2021.103178

Sehun, C., & Jihyun, L. (2021). Volatile and sensory profiles of different black rice (Oryza sativa L.) cultivars varying in milling degree. *Food Research International, 141*. doi: 10.1016/j.foodres.2021.110150

Zhan, S. J., Liu, Z. B., Su, W. Y., Lin, C. C., & Ni, L. (2023). Role of roasting in the formation of characteristic aroma of wuyi rock tea. Food Control, 147. doi: 10.1016/j.foodcont.2023.109614
